# Nerolidol Suppresses the Inflammatory Response during Lipopolysaccharide-Induced Acute Lung Injury via the Modulation of Antioxidant Enzymes and the AMPK/Nrf-2/HO-1 Pathway

**DOI:** 10.1155/2019/9605980

**Published:** 2019-11-16

**Authors:** Yung-Lun Ni, Huan-Ting Shen, Chun-Hung Su, Wen-Ying Chen, Rosa Huang-Liu, Chun-Jung Chen, Shih-Pin Chen, Yu-Hsiang Kuan

**Affiliations:** ^1^Department of Pulmonary Medicine, Taichung Tzu Chi Hospital, Buddhist Tzu Chi Medical Foundation, Taichung, Taiwan; ^2^Institute of Biochemistry, Microbiology, and Immunology, Chung Shan Medical University, Taichung, Taiwan; ^3^Department of Internal Medicine, School of Medicine, Chung Shan Medical University, Taichung, Taiwan; ^4^Department of Internal Medicine, Chung Shan Medical University Hospital, Taichung, Taiwan; ^5^Department of Veterinary Medicine, National Chung Hsing University, Taichung, Taiwan; ^6^School of Nutrition, Chung Shan Medical University, Taichung, Taiwan; ^7^Department of Education and Research, Taichung Veterans General Hospital, Taichung, Taiwan; ^8^Department of Pharmacology, School of Medicine, Chung Shan Medical University, Taichung, Taiwan; ^9^Department of Pharmacy, Chung Shan Medical University Hospital, Taichung, Taiwan

## Abstract

Acute lung injury (ALI) is a life-threatening disease that is characterised by the rapid onset of inflammatory responses. Lipopolysaccharide (LPS) is an endotoxin that plays an important role in triggering ALI via pneumonia and sepsis. However, no effective therapeutic strategies are currently available to treat ALI. Nerolidol is an aliphatic sesquiterpene alcohol that is found in the essential oils of many flowers as well as floral plants. It has been shown to exhibit anti-inflammatory, antioxidant, and anticancer properties. Herein, we show that nerolidol pretreatment counteracted the histopathological hallmarks in LPS-induced ALI mice. Indeed, nerolidol pretreatment inhibited LPS-induced alveolar-capillary barrier disruption, lung edema, and lipid peroxidation. Moreover, nerolidol pretreatment prevented the LPS from decreasing the enzymatic activities of superoxide dismutase, catalase, and glutathione peroxidase. Importantly, nerolidol treatment enhanced phosphorylation of AMP-activated protein kinase (AMPK) and expression of nuclear factor erythroid-derived 2-related factor 2 (Nrf-2) and heme oxygenase-1 (HO-1). Taken together, our study reveals the novel protective effects of nerolidol in LPS-induced ALI via the induction of antioxidant responses and activation of the AMPK/Nrf-2/HO-1 signalling pathway.

## 1. Introduction

Acute lung injury (ALI) is generally characterised by the rapid onset of inflammatory responses, including bilateral pulmonary neutrophil infiltration, haemorrhage, hyaline membrane formation, lung edema, and hypothermia [1]. In humans, ALI and acute respiratory distress syndrome (a more severe form of ALI) score highly in terms of morbidity and mortality rates worldwide [2, 3]. ALI can lead to the development of pneumonia as well as sepsis. However, no effective therapeutic strategies for ALI are currently available. Lipopolysaccharide (LPS) is a glucosamine-based saccharolipid and the main element of the outer lipid membrane in Gram-negative bacteria [4]. Consequently, LPS may play an important role in triggering pneumonia and sepsis [2].

In an animal experimental model, LPS instillation causes the activation of tissue-resident leukocytes and the recruitment of peripheral blood leukocytes to the lungs through the disrupted alveolar-capillary barrier [5–7]. The activation of leukocytes induces degranulation and a respiratory burst for the robust production of reactive oxygen species (ROS) such as superoxide anion, hydrogen peroxide, and hydroxyl radical [8]. In cells, the nuclear factor erythroid-derived 2-related factor 2 (Nrf-2)/heme oxygenase-1 (HO-1) pathway, as well as the activities of antioxidant enzymes (AOEs) such as superoxide dismutase (SOD), catalase (CAT), and glutathione peroxidase (GPx), are activated during oxidative stress. These enzymes catalyse chemical reactions to counteract ROS-induced oxidative damages, including lipid peroxidation and tissue damage [5, 9–11]. The nuclear accumulation and phosphorylation of Nrf-2 is facilitated by AMP-activated protein kinase (AMPK) signalling [10]. Interestingly, in murine ALI models, LPS has been shown to inactivate AMPK signalling and downregulate AOEs [12, 13].

Nerolidol (3,7,11-trimethyl-1,6,10-dodecatrien-3-ol) is an aliphatic sesquiterpene alcohol found in the essential oils of many flowers and plants with a floral scent. Nerolidol is present in neroli, ginger, citronella, lemongrass, rose, and tea tree [14, 15]. Despite the well-documented anti-inflammatory, antioxidant, antimicrobial, and anticancer properties of nerolidol [16], no studies have so far evaluated the protective effects as well as the molecular mechanisms of nerolidol on ALI. Herein, we report a previously uncharacterised protective role of nerolidol during LPS-induced ALI in mice that is associated with the AMPK/Nrf-2/HO-1 pathway and antioxidant responses.

## 2. Materials and Methods

### 2.1. Materials

Antibody against phospho-AMPK (catalog Number 2535) was acquired from Cell Signalling Technology, Inc. (Beverly, MA). Nerolidol and antibodies against AMPK (catalog Number SC-25792), Nrf-2 (catalog Number SC-13032), HO-1 (catalog Number SC-10789), and *β*-actin (catalog Number SC-47778) were acquired from Santa Cruz Biotechnology Inc. (Santa Cruz, CA). A thiobarbituric acid reactive substance (TBARS) assay kit, CAT assay kit, SOD assay kit, and GPx assay kit were obtained from Cayman Chemical Co. (Ann Arbor, MI). Lipopolysaccharide (LPS; *Escherichia coli*, serotype 0111:B4) and other reagents were purchased from Sigma-Aldrich (St. Louis, MO).

### 2.2. Mice and Experimental Design

Male BALB/c mice aged 8–10 weeks weighing 25–35 g were obtained from the National Laboratory Animal Center (Taipei, Taiwan). Mice were housed under a 12 : 12 h light-dark cycle with free access to a laboratory rodent diet. All animal experiments were conducted in accordance with the Institutional Animal Ethics Committee of Chung Shan Medical University. The mice were randomly divided into six groups as follows: control, LPS, nerolidol (10, 30, and 100 *μ*mol/kg)+LPS, and dexamethasone (1 mg/kg)+LPS groups. The control group received vehicle intraperitoneally (IP) for 30 min followed by the intranasal administration of 20 *μ*L saline by drops applied with a pipette. The LPS, nerolidol+LPS, and dexamethasone+LPS groups received vehicle, nerolidol, and dexamethasone IP for 30 min followed by the intranasal administration of LPS at 100 *μ*g/20 *μ*L of saline by drops applied with a pipette. Mice were euthanised by pentobarbital after LPS treatment for 24 h [7]. Bronchoalveolar lavage fluid (BALF) and lung tissues were collected. Bronchoalveolar lavage was collected by flushing 1 mL of sterile saline via the tracheal cannula three times. After the collection and centrifugation steps were completed, the protein concentrations were determined using the Bradford protein assay (Bio-Rad Laboratories) in a supernatant of BALF [7].

### 2.3. Lung Histopathology

The lungs were excised, soaked in 10% formalin, and embedded in paraffin. Tissue blocks were sectioned into 4 *μ*m thick sections using the rotary microtome. Sections were stained with hematoxylin-eosin. Under a light microscope, the level of histopathological changes was evaluated by leukocyte infiltration, alveolar wall thickness, and hyaline membrane formation in a blind manner by 50 microscopic fields randomly [5].

### 2.4. Wet-to-Dry Lung Weight Ratio

The index of lung edema after LPS administration was measured using a wet-to-dry (W/D) weight ratio [6], obtained by the weight measured immediately after excision (wet) and the weight after dehydration for 48 h at 80°C (dry).

### 2.5. Myeloperoxidase (MPO) Assay

Twenty-four hours after LPS administration, the lungs were excised and homogenised in MPO extractive phosphate buffer containing guaiacol and cetyltrimethylammonium bromide. After being subjected to centrifugation, the supernatant was reacted with hydrogen peroxide. The levels of MPO were indicated by absorbance at 470 nm [5].

### 2.6. Lipid Peroxidation Assay

The presence of malondialdehyde (MDA) and the production of lipid peroxidation were evaluated in the lungs using the TBARS assay kit following the manufacturer's protocol as described previously [5]. The lung homogenate was incubated with thiobarbituric acid reactive substances, including thiobarbituric acid and trichloroacetic acid. The chromogenic reaction was carried out at 100°C for 1 h, and the absorbance was measured at 530 nm.

### 2.7. Antioxidative Enzyme (AOE) Assay

The activities of antioxidative enzymes (AOEs), including SOD, CAT, and GPx, were measured using commercial assay kits for SOD, CAT, and GPx, respectively, according to the manufacturer's instructions [5].

### 2.8. Western Blot Assay

The total proteins in the harvested lungs were homogenised and extracted using the T-PER solution (Pierce, Rockford, IL). Equal amounts of protein were separated using SDS-PAGE at 7.5% and transferred onto PVDF membranes. Membranes were blocked using 5% nonfat milk in phosphate-buffered saline containing 0.1% Tween-20 for 1 h at room temperature, and probed with primary antibodies including phospho-AMPK, AMPK, Nrf-2, HO-1, and *β*-actin. Membranes were washed and incubated with horseradish peroxidase- (HRP-) labelled secondary antibodies for 1 h. The membranes were detected using ECL Plus Western blotting detection reagents [6].

### 2.9. Statistical Analysis

In this study, at least three separate repetitions of each experiment were performed. Unless otherwise specified, the data are presented as mean ± standard deviation. Statistical analyses were performed using SPSS 14.0 statistical software (SPSS, Chicago, IL). One-way analysis of variance and the Bonferroni *t*-test for multigroup comparisons were used to calculate the *P* values. *P* < 0.05 was considered statistically significant.

## 3. Results

### 3.1. Nerolidol Protects against LPS-Induced ALI

**Figure 1:**
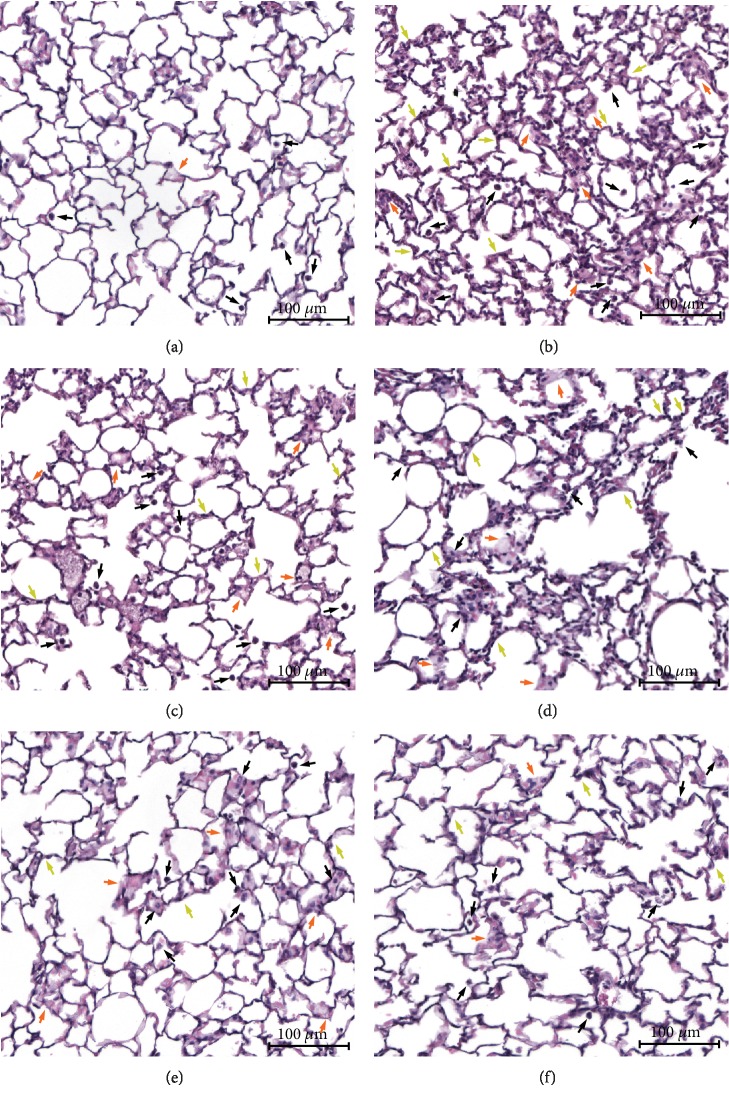
Nerolidol protects against the histopathological exchange of lungs in LPS-induced ALI. (a) Control, (b) LPS, (c) 10 *μ*mol/kg nerolidol+LPS, (d) 30 *μ*mol/kg nerolidol+LPS, (e) 100 *μ*mol/kg nerolidol+LPS, and (f) 1 mg/kg dexamethasone+LPS. Hematoxylin-eosin staining of lung sections of each experimental group (magnification: ×100; scale bars represent 100 *μ*m). Black arrow, neutrophil infiltration; orange arrow, haemorrhage and hyaline membrane formation; green arrow, alveolar wall thickness and edema.

To evaluate the protective effects of nerolidol on acute pulmonary inflammation, the murine model of LPS-induced ALI was implemented. Thirty minutes after the IP administration of nerolidol at differential concentrations, the mice were subjected to intranasal instillation with either saline (control) or LPS. After 24 h, we observed normal pulmonary structures and no histopathological changes using light microscopy in the control group (Figure 1(a)). As expected, we observed neutrophil infiltration, alveolar wall thickening, haemorrhage, and hyaline membrane formation after LPS administration (Figure 1(b)). Notably, nerolidol pretreatment alleviated the LPS-mediated histopathological hallmarks in a dose-dependent manner (Figures 1(c)–1(e)). The LPS-induced histopathology was also reduced in the presence of dexamethasone, which is an anti-inflammatory steroid (Figure 1(f)), suggesting that nerolidol exhibited anti-inflammatory effects in the lungs during LPS-induced ALI.

### 3.2. Nerolidol Protects against LPS-Induced Alveolar-Capillary Barrier Disruption and Leukocyte Infiltration

**Figure 2:**
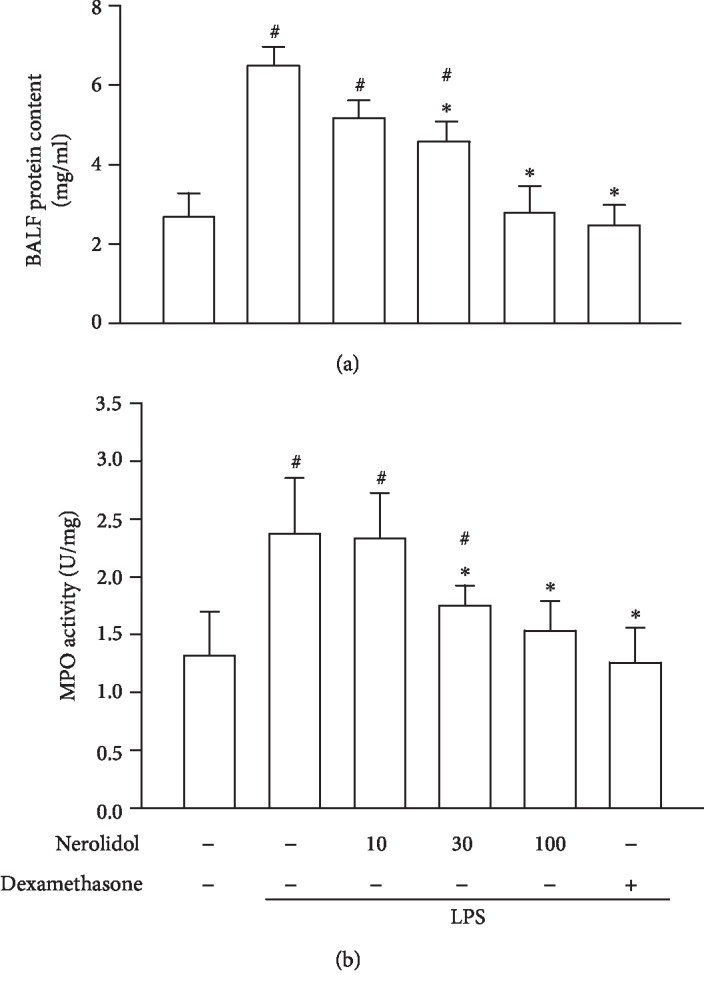
Nerolidol protects against LPS-induced alveolar-capillary barrier disruption and leukocyte infiltration. (a) Alveolar-capillary barrier disruption was determined by protein leakage via a Bradford protein assay. (b) Leukocyte infiltration was determined by MPO activity in BALF. Values are expressed as mean ± S.D. of 3-4 mice per group. ^#^*P* < 0.05 represents a significant difference between the indicated group and the control group; ^∗^*P* < 0.05 represents a significant difference between the indicated group and the LPS groups.

Neutrophil activation and infiltration play an essential role in LPS-induced ALI. Alveolar-capillary barrier disruption by activated neutrophils and LPS results in plasma protein and neutrophil leakage into the alveolar space [7]. We found that LPS-treated mice exhibited a significantly higher protein concentration in the BALF compared with control mice (*P* < 0.05). However, this was significantly reduced in mice pretreated with nerolidol at a concentration of 30 *μ*mol/kg; the protein concentration in BALF was also significantly reduced (*P* < 0.05; Figure 2(a)). Furthermore, we found that while the MPO content in the lungs increased significantly after LPS administration (*P* < 0.05), this was significantly inhibited by nerolidol pretreatment at 30 *μ*mol/kg (*P* < 0.05; Figure 2(b)), suggesting that the activation and recruitment of neutrophils were suppressed by nerolidol.

### 3.3. Nerolidol Protects against LPS-Induced Lung Edema

**Figure 3:**
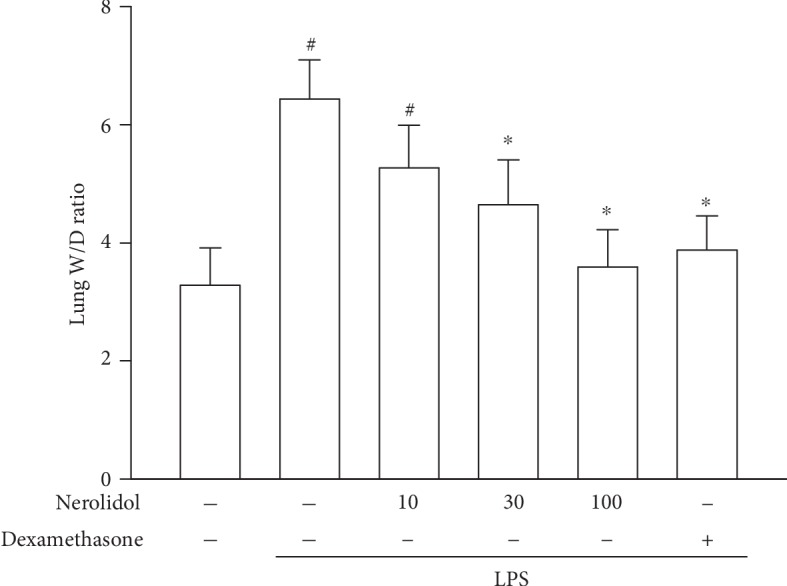
Nerolidol protects against LPS-induced lung edema. Lung edema was determined by the W/D ratio. Values are expressed as mean ± S.D. of 3-4 mice per group. ^#^*P* < 0.05 represents a significant difference between the indicated group and the control group; ^∗^*P* < 0.05 represents a significant difference between the indicated group and the LPS groups.

Lung edema, an excess accumulation of fluid in the lungs, is caused by the disruption of the alveolar-capillary barrier and is a critical pathological feature in ALI [6]. By characterising the wet and dry weights of the lungs (see Materials and Methods), we found that LPS-treated mice exhibited increased W/D ratio compared with control mice (*P* < 0.05), indicating the manifestation of lung edema in these mice. Similar to our previous observation, pretreatment with nerolidol inhibited LPS-induced lung edema in a dose-dependent manner; a significant effect started at 30 *μ*mol/kg (*P* < 0.05; Figure 3).

### 3.4. Nerolidol Protects against LPS-Induced Lipid Peroxidation in the Lungs

**Figure 4:**
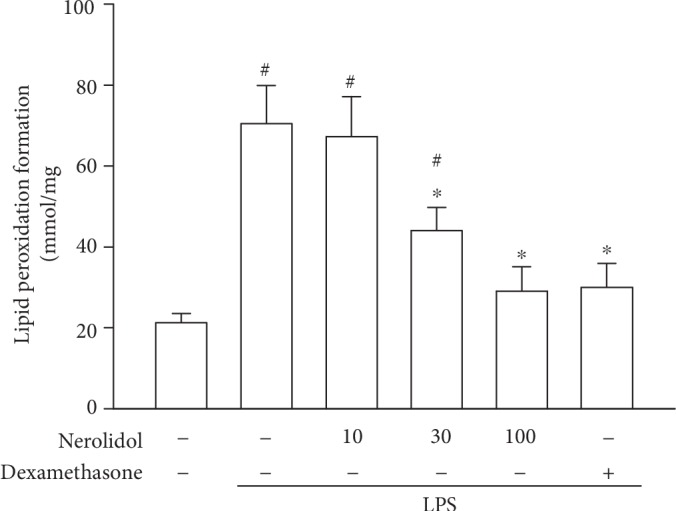
Nerolidol protects against LPS-induced lipid peroxidation in the lungs. Lipid peroxidation was determined by the MDA formation. Values are expressed as mean ± S.D. of 3-4 mice per group. ^#^*P* < 0.05 represents a significant difference between the indicated group and the control group; ^∗^*P* < 0.05 represents a significant difference between the indicated group and the LPS groups.

Leukocyte activation and infiltration result in lipid peroxidation, which is a critical risk factor in the pathogenesis of ALI [5]. MDA is the product of lipid peroxidation and can be used as an indicator for lipid peroxidation rate. We observed that while LPS treatment led to a significant increase in MDA levels in mice (*P* < 0.05), this effect was significantly inhibited by nerolidol pretreatment in a dose-dependent manner; a significant effect started at 30 *μ*mol/kg (*P* < 0.05; Figure 4), suggesting that nerolidol pretreatment could effectively suppress lipid peroxidation induced by ALI-mediated leukocyte infiltration.

### 3.5. Nerolidol Counteracts LPS-Mediated AOE Inhibition

**Figure 5:**
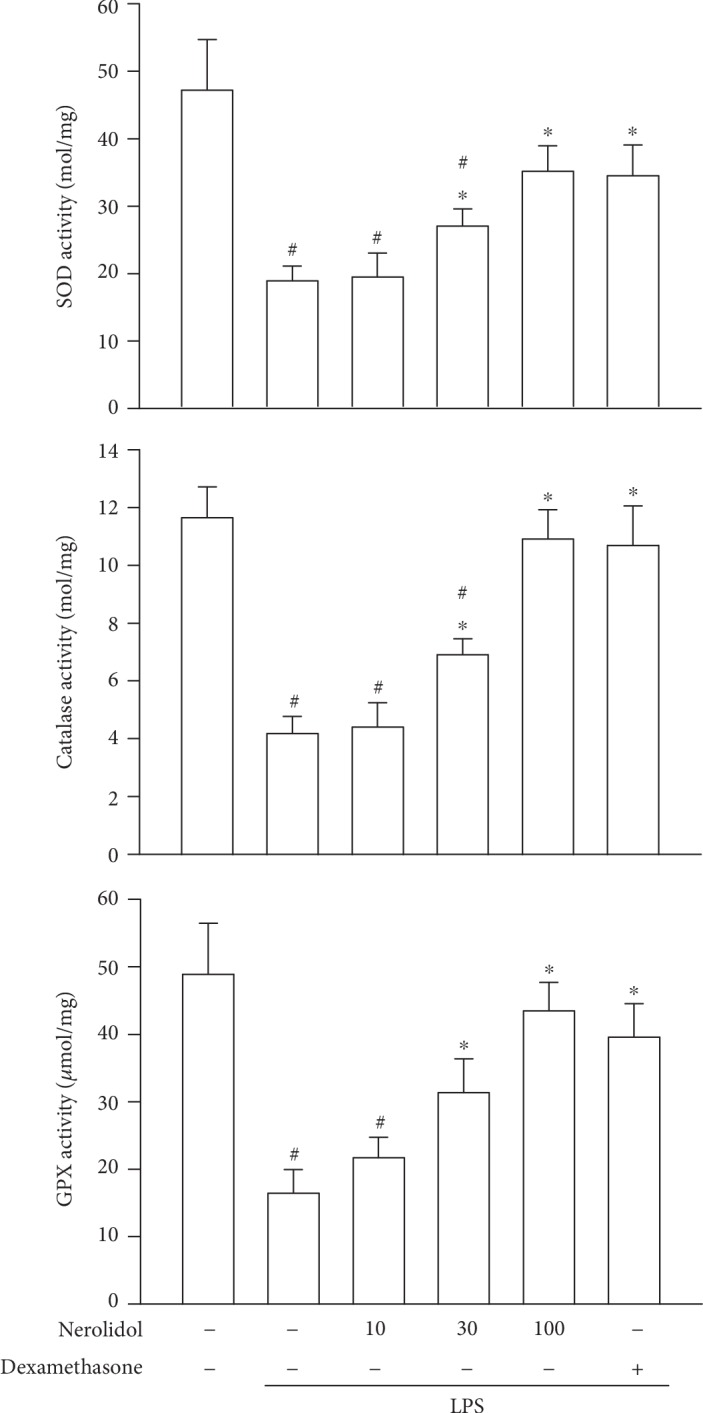
Nerolidol protects against LPS-reduced AOE activities. The AOEs represented are SOD, CAT, and GPx. Values are expressed as mean ± S.D. of 3-4 mice per group. ^#^*P* < 0.05 represents a significant difference between the indicated group and the control group; ^∗^*P* < 0.05 represents a significant difference between the indicated group and the LPS groups.

Given the seemingly potent anti-inflammatory efficacy, we next asked how nerolidol pretreatment might exert such effects. Oxidative stress that facilitates lipid oxidation can be opposed by the activities of AOEs, such as SOD, CAT, and GPx [5]. We noted that the activities of SOD, CAT, and GPx were suppressed in LPS-treated mice compared with control mice (*P* < 0.05; Figure 5), indicating that LPS-induced ALI and leukocyte recruitment were associated with decreased AOE activities. Importantly, we found that nerolidol pretreatment was sufficient to prevent the decrease of AOE activities in a dose-dependent manner; a significant effect started at 30 *μ*mol/kg (*P* < 0.05; Figure 5), indicating that nerolidol might reduce inflammatory tissue damage via the upregulation of the antioxidant response.

### 3.6. Nerolidol Prevented the LPS-Induced Repression of Nrf-2 and HO-1 Expression

**Figure 6:**
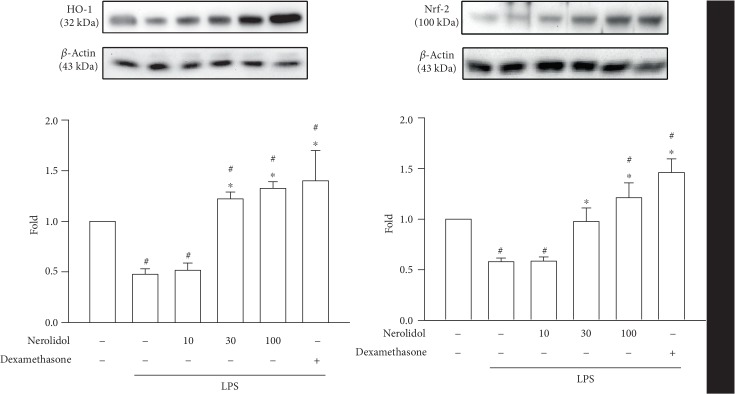
Nerolidol enhances LPS-induced Nrf2 and HO-1 expression. The lung lysates were analyzed by Western blotting. Values are expressed as mean ± S.D. of 3-4 mice per group. ^#^*P* < 0.05 represents a significant difference between the indicated group and the control group; ^∗^*P* < 0.05 represents a significant difference between the indicated group and the LPS groups.

HO-1 is an antioxidative protein involved in the resolution of inflammation. Its expression is regulated by the transcription factor Nrf-2 [5]. We found that both Nrf-2 and HO-1 expressions were significantly reduced in LPS-treated mice compared with control mice (*P* < 0.05), indicating that LPS treatment was associated with the downregulation of the Nrf-2/HO-1 transcription response. Importantly, pretreatment with nerolidol prevented the LPS-induced repression of Nrf-2 and HO-1 and enhanced LPS-reduced repression in a dose-dependent manner; a significant effect started at 30 *μ*mol/kg (*P* < 0.05; Figure 6), suggesting that nerolidol-mediated protective response involved the Nrf-2/HO-1 transcription axis.

### 3.7. Nerolidol Prevented the LPS-Induced Repression of AMPK Phosphorylation

**Figure 7:**
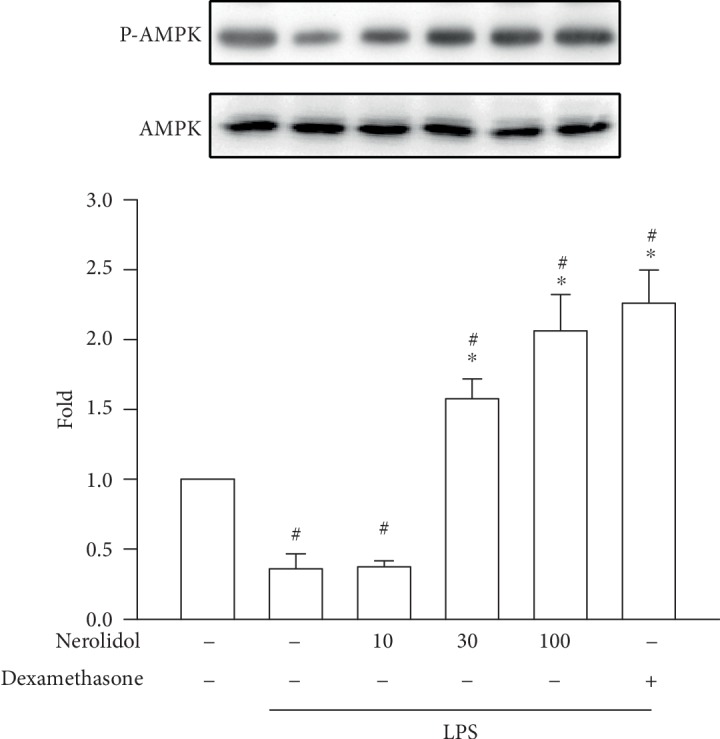
Nerolidol enhances LPS-induced AMPK phosphorylation. The lung lysates were analyzed by Western blotting. Values are expressed as mean ± S.D. of 3-4 mice per group. ^#^*P* < 0.05 represents a significant difference between the indicated group and the control group; ^∗^*P* < 0.05 represents a significant difference between the indicated group and the LPS groups.

AMPK signalling induces the nuclear accumulation of Nrf-2 [17]. To determine whether the effects on Nrf-2 during LPS-induced ALI were dependent on AMPK activity, we measured AMPK phosphorylation using our experimental setup. Consistent with our hypothesis, we found that the phosphorylation of AMPK was significantly reduced in LPS-treated mice compared with control mice (*P* < 0.05), suggesting that LPS-induced ALI was associated with the downregulation of AMPK signalling. Notably, nerolidol pretreatment inhibited LPS-induced AMPK silencing and enhanced phosphorylation of AMPK in a dose-dependent manner; a significant effect started at 30 *μ*mol/kg (*P* < 0.05; Figure 7).

## 4. Discussion

Nerolidol is the sesquiterpene compound found in essential oils from flowers and plants [14, 15]. Nerolidol is widely used as a fragrant ingredient and flavouring and as a fixative reagent in detergents and perfumes [18, 19]. It is also used in many food products as a flavour enhancer, and its use is permitted by both the United States Food and Drug Administration and the European Food Safety Authority [20]. In addition, nerolidol has several pharmacological effects, such as anti-inflammatory and antioxidant activities. In a previous study, nerolidol at concentrations of 100 and 200 mg/kg has been shown to suppress LPS-induced acute kidney inflammation in rat models [21]. Moreover, nerolidol reduces the generation of proinflammatory mediators in LPS-activated peritoneal macrophages [22]. ALI is the pulmonary disorder of acute inflammation directly induced by LPS instillation in mouse models [22]. LPS instillation causes apparent histopathology including neutrophil infiltration, haemorrhage, hyaline membrane formation, and lung edema [1]. These pathological characteristics are similar to clinical signs in ALI patients [23]. In this study, the histopathological results of LPS-induced ALI correlated well with those of previous studies [1, 6]. In addition, we showed that the LPS-induced histopathological characteristics of ALI could be counteracted by nerolidol *in vivo* in a dose-dependent manner. Importantly, these results indicated that LPS-induced inflammatory responses in ALI could be reversed by nerolidol.

Leukocytes, especially neutrophils, are the prime culprit in the pathogenesis of ALI. After the administration of LPS, proinflammatory mediators from alveolar macrophages and pulmonary cells stimulate neutrophil activation within peripheral circulation [6]. Migration, respiratory burst, and degranulation of neutrophils are critical to the innate immune system [24]. Essential oil extracted from *Peperomia serpens* Loud (EOP), which mainly contains nerolidol, has been shown to modulate acute inflammatory responses through the interference of leukocyte migration, rolling, and adhesion in rodent models [25]. EOP inhibits the paw edema induced by carrageenan and dextran, as well as the ear edema induced by croton oil [25]. Consistent with previous data, our results suggested that LPS-induced neutrophil infiltration into the lungs and the disruption of the alveolar-capillary barrier could be reduced by nerolidol, as revealed by MPO and protein leakage assays, respectively. Moreover, lung edema was also ameliorated by nerolidol in our LPS-induced ALI model. These results indicated that nerolidol reduced LPS-induced lung edema through inhibiting neutrophil infiltration and alveolar-capillary barrier disruption.

During inflammation, leukocytes produce large quantities of ROS in response to an invasive pathogen. While exhibiting antimicrobial effects, excessive oxidative stress can also cause injury to peripheral tissues. Lipid peroxidation is the most common readout of oxidative stress and potential tissue damage [26]. Studies have indicated that nerolidol inhibits lipid peroxidation in rotenone-induced midbrain tissue damage and *Trypanosoma evansi*-mediated brain and hepatic damage [27–29]. In this current study, we found that lipid peroxidation induced by LPS in the lungs could be inhibited by nerolidol in a dose-dependent manner. In cells, oxidative stress, or the level of ROS, is generally counterbalanced by antioxidant responses. These include the AOEs and signalling pathways such as the Nrf-2/HO-1 axis [9, 10]. It has been shown that ROS generation reduces the capacities of AOEs, including SOD, CAT, and GPx [5]. Interestingly, rotenone- or *Trypanosoma evansi*-induced AOE suppression could be reversed by nerolidol in the brain or liver [27–29]. Herein, we show that pretreatment with nerolidol significantly prevented the decrease of AOE activities in the lungs of mice treated with LPS using our ALI model. These results suggested that nerolidol prevented neutrophil-associated tissue damage likely through the reduction of lipid peroxidation and upregulation of AOE activities during LPS-induced ALI in mice.

Nrf-2 is an important transcription factor and the master regulator of antioxidant defence molecules [30]. The ROS-mediated activation of Nrf-2 induces the expression of many different proteins, including HO-1, the phase-II detoxifying and antioxidative protein. The activation, nuclear translocation, and stabilisation of Nrf-2 are enhanced by 3S-(+)-9-oxonerolidol, a derivative of nerolidol, in human lung epithelial cells [31]. In the present study, we showed that LPS inhibited the expression of Nrf-2 and HO-1 and that these were rescued and further enhanced by nerolidol in a dose-dependent manner. In addition, our results indicated that oxidative stress induced the activation and expression of Nrf-2 through the activation of AMPK signalling, which is a crucial player during various inflammatory and oxidative stresses [17]. As shown in our results, we found that the level of AMPK phosphorylation in the presence of nerolidol was positively correlated with the expression of Nrf-2 in LPS-treated mice. These results further indicated that HO-1 expression was likely influenced by the AMPK/Nrf-2 pathway in our model.

**Figure 8:**
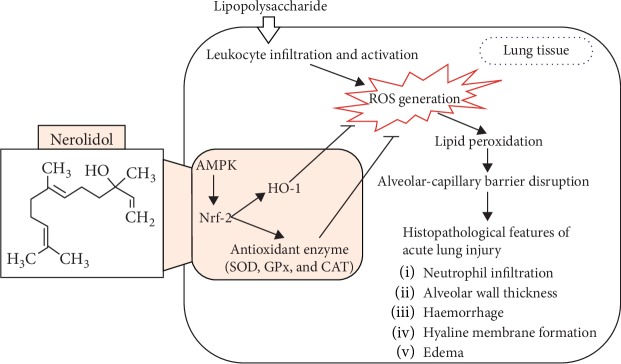
Scheme of the mechanisms in the protective effect of nerolidol on LPS-induced ALI.

In conclusion, we have demonstrated that nerolidol pretreatment effectively protected against the exacerbation of lung histopathology, including neutrophil infiltration, alveolar wall thickening, haemorrhage, hyaline membrane formation, and edema, during LPS-induced ALI. The protective mechanisms of nerolidol include (1) the inhibition of alveolar-capillary barrier disruption and leukocyte infiltration; (2) the reduction of lipid peroxidation; (3) the prevention of AOE activities; and (4) the prevention of the decrease and increase of HO-1 expression, Nrf-2 expression, and AMPK phosphorylation. Taken together, our findings suggested that the beneficial effects of the application of nerolidol in the prevention of ALI inflammation most likely involves the restoration of the AMPK/Nrf-2/HO-1 pathway and AOE activities (model illustrated in Figure 8). The protective effects of nerolidol are similar as the monocyclic sesquiterpene derivative, zerumbone, on LPS-induced ALI mice. A previous study has proposed that the protective mechanism of zerumbone on LPS-induced ALI was via the upregulation of AOEs and the Nrf-2/HO-1 pathway [5]. This evidence could be used to propose that ALI could be reduced by sesquiterpene derivatives, including nerolidol and zerumbone, via the restoration of the AOE activities, HO-1, and the relative upstream pathway.

## Data Availability

The data of this manuscript entitled “Nerolidol Suppresses the Inflammatory Response during Lipopolysaccharide-Induced Acute Lung Injury via the Modulation of Antioxidant Enzymes and the AMPK/Nrf-2/HO-1 Pathway” (manuscript No. 9605980) is under license and so cannot be made freely available. Requests for access to these data should be made to Yu-Hsiang Kuan through the following E-mail address: kuanyh@csmu.edu.tw.
